# Factors related to the amount of energy required for weight gain in patients with anorexia nervosa under strict behavioral control: a study in a Japanese medical prison

**DOI:** 10.1186/s13030-025-00332-0

**Published:** 2025-07-01

**Authors:** Chie Aso Suzuyama, Shu Takakura, Masato Takii, Junji Kishimoto, Kenta Toda, Makoto Yamashita, Tomokazu Hata, Nobuyuki Sudo

**Affiliations:** 1 Department of Psychosomatic Medicine, Graduate School of Medical Sciences, Kyushu University, 3-1-1 Maidashi, Higashi-Ku, Fukuoka-Shi, Fukuoka, 812-8582 Japan; 2 Department of Psychosomatic Medicine, Kyushu University Hospital, 3-1-1 Maidashi, Higashi-Ku, Fukuoka-Shi, Fukuoka, 812-8582 Japan; 3 Center for Clinical and Translational Research, Kyushu University, 3-1-1 Maidashi, Higashi-Ku, Fukuoka-Shi, Fukuoka, 812-8582 Japan; 4 Kitakyushu Medical Prison, 1-1-1 Hayama-Cho, Kokuraminami-Ku, Kitakyushu-Shi, Fukuoka, 802-0837 Japan; 5 Fukuoka Tokushukai Hospital, 4-5 Sugukita, Kasuga-Shi, Fukuoka, 816-0864 Japan

**Keywords:** Anorexia nervosa, Excess energy, Weight gain, Body composition, Multifrequency bioelectrical impedance

## Abstract

**Background:**

Weight restoration is a crucial factor in the treatment of anorexia nervosa (AN); however, there is currently no consensus on the amount of energy required to achieve weight gain in patients with AN.

**Methods:**

Forty-one patients with AN in a Japanese medical prison were included in the study. All the data were collected from their medical charts. Body weight and composition were measured using the multifrequency bioelectrical impedance device InBody 770®. Daily caloric intake was determined by subtracting the amount of calories in leftover food from that provided, and resting energy expenditure was calculated using Scalfi's formulation. These were then used to calculate daily amount of energy and energy necessary for a 1-kg weight gain (EE1). In addition, we investigated the relation between EE1 and the initial body composition values, body mass index, and AN subtype.

**Results:**

Of the 41 patients, two had EE1s that we considered outliers. Excluding these, the mean EE1 was 12,776.8 kcal/kg, with large individual differences observed (range: 6,636–22,064 kcal/kg). Significant associations were noted between EE1 and body fat mass, body fat percentage, soft lean percentage, and body water ratio. Moreover, patients with body fat mass (BFM) ≤ 3 kg (*p* = 0.003), body fat percentage (BFP) ≤ 8% (*p* = 0.008), soft lean percentage (SLP) > 85% (*p* = 0.011), and body water ratio (BWR) > 0.665 (*p* = 0.011) had higher mean EE1s.

**Conclusions:**

To gain weight, patients with AN may require a higher caloric intake than that reported in the literature for healthy women, particularly patients with less fat and more muscle. (242/350words).

## Background

Anorexia nervosa (AN) is a mental illness characterized by excessive calorie restriction and fear of weight gain that can be fatal [1]. Moreover, concerns regarding body shape and weight lead to excessive weight loss, which may cause other physical and mental comorbidities [2, 3] that may prolong and complicate the disease, making it more difficult to treat [4]. Therefore, renourishment and weight recovery are the most important aspects of AN treatment [5–7].

While many aspects of the refeeding of patients with AN have been elucidated, the number of calories required to gain weight remains unclear. The 2004 National Institute for Health and Care Excellence (NICE) guidelines recommended a weight gain of 0.5 kg to 1.0 kg per week, requiring approximately 3,500 kcal to 7,000 kcal extra calories per week [7]. However, other reports have included a wider range of calories needed for a weight gain of 1 kg [8].

A higher caloric intake is required for an increase in weight of 1 kg in patients with AN than that required for the same weight increase in healthy women or patients with bulimia nervosa [9–11], despite the low body weight of patients with AN. This finding may be explained by changes in gut microbiota or function [12, 13], changes in endocrine function [14], elevated diet-induced thermogenesis (DIT) [15, 16], or hyperactivity, which is an AN-specific behavior. We hypothesized that the excess energy intake required to gain 1 kg of body weight during the weight recovery period for women with AN would be more than that required for healthy women. Additionally, behaviors commonly observed in patients with AN, such as food disposal, as well as self-induced vomiting observed in those with the binge-eating/purging subtype (AN-BP), may also have influenced the findings of previous studies. Although weight gain in patients with AN is important for recovery, the reported calories required for weight gain have been inconsistent. Furthermore, most previous reports have included study populations of 20 or fewer patients, which may not be sufficient for conclusive results.

Concerns regarding body shape and weight are important for patients with AN, increasing resistance to body shape and weight changes induced by refeeding [2]. In our practice, we often encounter patients with an increased fear of obesity due to unexpected weight gain who conceal vomiting or food wasting, even during inpatient treatment. In such cases, knowledge of the EE1 may allow patients to better cope with their fear of obesity, as they gain awareness of the process of weight gain. Therapists can also use the EE1 as a reference for formulating the nutrition regimen and as guidance for deciding whether the patient's weight gain from the nutrition program is appropriate. Therefore, the ability to predict the amount of weight gain and possible body changes may facilitate recovery from AN. Furthermore, strict management of patient behavior in studies on energy intake for weight gain in patients with AN may also be required. Previous studies have been conducted in general hospital settings, where strictly managing patient behavior may have been difficult.

The present study aimed to investigate how many calories were required for a weight gain of 1 kg in the recovery period of patients with AN and to identify factors influencing weight gain. Metabolic rates differ between body fat mass (BFM) and fat-free mass (FFM). FFM includes organs with high metabolic activity, such as the liver, brain, and kidneys, which contribute significantly to resting energy expenditure (REE). In contrast, BFM has minimal metabolic impact. Therefore, we hypothesized that pre weight-recovery body composition, particularly the ratio of BFM to FFM, influences the amount of energy required for weight gain [17][18].

## Methods

### Participants

This study was a retrospective, observational study. Data were collected at Kitakyushu Medical Prison and analyzed at the Department of Psychosomatic Medicine, Kyushu University.

The study enrolled 41 female patients with AN who were imprisoned and treated at Kitakyushu Medical Prison between February 2013 and July 2018. The diagnosis of AN was according to the Diagnostic and Statistical Manual of Mental Disorders-5 [19]. Patients whose first anthropometric measurements were taken more than 30 days after admission or who experienced severe physical complications causing weight loss for reasons other than AN were excluded from the study. Specifically, one patient was excluded due to a prolonged fasting period following abdominal surgery.

This study was approved by the Kitakyushu Medical Prison Ethics Review Committee and the Kyushu University Clinical Research Ethics Review Committee[22013–00]. The ethical aspects and the necessity of the study involving prisoners were reviewed by both ethics committees in accordance with the principles of the Declaration of Helsinki. All the data were collected from the medical charts of the patients enrolled, all of whom were treated in the Kitakyushu Medical Prison. We were unable to contact the patients after their release from prison to obtain direct consent; however, we announced the study on the bulletin board of Kitakyushu Medical Prison and on the website of the Department of Psychosomatic Medicine Graduate School of Medical Science Kyushu University, which made it possible for the patients to refuse participation in the study. Both ethics review committees waived the requirement for participant consent based on the retrospective study design and the anonymization and de-identification of data.

### Treatment

Initial treatment included nourishment and stabilization of the patient’s condition. Patients were started on a diet of approximately 30 kcal/kg/day (at least 1,000 kcal/day). We introduced a liquid nutritional supplement (100 kcal/day) containing phosphates and other trace elements for approximately 1 month to prevent refeeding syndrome [20, 21]. The caloric intake provided did not exceed 2,100 kcal/day—an intake of 1,800–2,100 kcal/day is recommended for healthy Japanese women. Initially, patients were only allowed to lie down in their rooms until their eating habits and condition had stabilized. Patients received an amount of food they could eat completely without effort, and liquid meals were added as supplements for those who were unable to eat an adequate amount of solid food to achieve an energy intake of 30 kcal/kg/day. Intravenous infusion was administered to 13 patients, accounting for 1.2% of the total caloric intake during the observation period. Notably, 95% of the infused calories were administered to only three patients. No patients received nasogastric tube feeding. We did not define the timeframe for administering meals with a specific calorie content. We also did not prescribe increments in activity or food intake with time. If the patients could easily consume all the food provided, we increased the amount by 200 kcal. However, if patients could not finish a meal or experienced vomiting, we reduced their food intake and substituted it with a liquid meal to ensure adequate energy intake. We controlled these aspects to maintain weight gain until the patient achieved a healthy weight, defined as a BMI exceeding 18.5 kg/m^2^ [22]. Our psychobehavioral treatment is based on a"cognitive-behavioral approach with behavioral limitation"[20]. This treatment incorporates the techniques of operant conditioning therapy and cognitive behavioral therapy. The primary objective of this treatment is to facilitate the transformation of the patient's conventional coping behaviors, such as avoiding stressful situations and negative emotions through anorexia, overeating, and vomiting, into adaptive cognitive-behavioral patterns, thereby fostering mental growth. During the initial imprisonment phase, participants were only allowed to be active in their rooms. After this phase, they were allowed to expand their activities based on subsequent conditions, such as eating complete meals, quitting vomiting, or engaging in introspection and discussing it with their therapist. Finally, they were allowed up to two hours of sedentary light work and 30 min of outside activity per day. During outside activities, most participants read or walked slowly and did not engage in strenuous exercise. We did not use activity meters during our observations; therefore, we could not assess the exact amount of physical activity.

Particularly during the first half of the treatment, we emphasized the importance of patients being able to consume their meals completely and to stop vomiting. As the treatment progressed, this behavioral limitation was gradually eased in response to appropriate eating behaviors and weight regain. This approach has been shown to be effective in increasing weight and enabling patients to engage in self-reflection [20]. By eliminating the inappropriate avoidance behaviors previously adopted, the therapeutic structure allowed patients to confront their original negative emotions and emotional problems. In addition, patients attended sessions approximately once a week with an eating disorder specialist that included psychological interviews. Family interviews were held if considered necessary. Some patients who showed progress in their treatment and became capable of self-reflection were allowed to participate in group meetings.

Due to the nature of the medical prison facility, strict monitoring of the participants was in place, making self-induced vomiting and hiding or disposing of food nearly impossible. Except for work hours or outside activity hours, the participants were not allowed to leave their room without permission. Work and activities were monitored by prison officers. Furthermore, a monitoring camera is installed in each room and the patients use a toilet that they cannot flush by themselves. Moreover, access to water is monitored by prison officers, thus the participants could not drink without permission.

### Anthropometry

Body composition was assessed with the multifrequency bioelectrical impedance analysis (BIA) device InBody 770® (Biospace, Seoul, Korea), which can measure body weight (BW), fat, soft lean mass (SLM), and water values and can distinguish between total, trunk, and limb values. Patient body composition and body weight were measured by the BIA device once a week from admission to week 6, once every two weeks in weeks 7–12, and once a month after week 13. Measurements were taken at 8:30 am with patients wearing their uniforms and checked by a nurse or prison officer.

### Calculation of the amount of energy required to gain weight

After each meal, the prison officer or nurse checked the amount of food left on the tray. Main and side dishes were categorized separately, and the daily energy intake was calculated subtracting the calories in the remaining food from that of the food presented. The energy provided via liquid meals or intravascular nutrition was also added to the daily energy intake for patients requiring liquid nutritional supplementation. Daily excess energy (EE) was calculated daily using the latest BW measured by a BIA device.

For excess energy calculations, we used the Scalfi’s formulation [23]. The Scalfi’s formulation is used to calculate the resting energy expenditure of women with AN. The"0.238"in the formulation is the number used to convert units from"kJ"to"kcal. To calculate excess energy, the activity factor was set at 1.3 because the participants were only doing sedentary work for up to 2 h and light outdoor activities for up to 30 min per day. We calculated the excess energy as follows:

$$\mathrm{Scalfi}'s\;\mathrm{formulation}:\mathrm{resting}\;\mathrm{energy}\;\mathrm{expenditure}\left(\mathrm{kcal}/\mathrm{day}\right)=96.3\ast\mathrm{BW}\left(\mathrm{kg}\right)\ast0.238$$

$$\mathrm{Excess}\;\mathrm{Energy}\left(\mathrm{EE}:\mathrm{kcal}/\mathrm{day}\right)=\left[\mathrm{Intake}\;\mathrm{energy}\right]-\left[\mathrm{Scafi}'s\;\mathrm{resting}\;\mathrm{energy}\;\mathrm{expenditure}\right]\ast1.3$$

In this study, we defined a period during which the patient maintains or decreases their body weight for more than two months as a “plateau period” and the period before the “plateau period” as a “weight gain period.” We used the weight gain period only to calculate the energy required to gain weight. We calculated a regression line using the amount of changing BW as the explanatory variable and the accumulated excess energy as the objective variable for each patient. The coefficient of the regression equation indicated the excess energy required to increase 1-kg BW (EE1), after which we calculated the average EE1.

### Study of the factors for weight gain

We evaluated the relation between the first measurements of body composition, AN subtype, BMI, and EE1 by a scatter plot. Factors that showed correlations with EE1 were divided into two groups within the range of 25%–75% quartiles, and values showing a difference between the groups were investigated.

#### Statistical analysis

To investigate the factors for weight gain, a one-way analysis of variance was used to compare EE1 with categorical variables (subtype). The relation between EE1 and continuous variables (BMI and body composition) was analyzed using scatter plots and Pearson’s correlation coefficients. We used *t*-tests for between group comparisons of EE1 with continuous variables (BMI and body composition), and *p*-values of < 0.05 were considered statistically significant. Additionally, correlation coefficients of 1 >|r|≥ 0.7, 0.7 >|r|≥ 0.4, 0.4 >|r|≥ 0.2, and 0.2 >|r| indicated strong, moderate, weak, or no correlation, respectively [24]. All statistical analyses were performed with JMP Pro Ver.14 (SAS Institute Inc.).

## Results

### Clinical characteristics

A total of 64 patients with a diagnosis of AN were detained in the Kitakyushu Medical Prison between February 2013 and July 2018. Of these, 23 were excluded from the study because anthropometric measurements were first taken after more than 30 days of imprisonment. The baseline clinical characteristics of the patients enrolled and the changes in weight and BMI are presented in Table 1**.**

**Table 1:** Baseline characteristics and changes in the weight gain period

Subtype	AN-R	n = 10	
	AN-BP	n = 31	
Age			44.05 ± 8.38 (30–68)
Height (cm)			156.76 ± 6.44 (144.4–178.0)
Weight (kg)			32.22 ± 3.80 (25.9–40.4)
BMI (kg/m2)			13.12 ± 1.40 (9.9–15.8)
Total body water (L)			22.03 ± 2.64 (17.6–29.1)
Body water ratio			0.685 ± 0.037 (0.583–0.723)
ECW/TBW			0.394 ± 0.008 (0.376–0.407)
Body fat mass (kg)			2.30 ± 1.53 (0.8–6.2)
Body fat percentage (%)			7.04 ± 4.55 (3–20.4)
Soft lean mass (kg)			28.18 ± 3.41 (22.4–37.2)
Weight gain period (days)			302 ± 108 (97–531)
Weight change (kg)			14.94 ± 5.32 (2.5–24.5)
BMI change (kg/m2)			6.1 ± 2.2 (0.9–9.8)
Daily calorie intake(kcal/day)			1858 ± 278 (50–2560)
Final weight(kg)			47.2 ± 6.2 (33.9–57.1)
Final BMI(kg/m^2^)			19.2 ± 2.5 (13.2–23.6)

Values are means ± SD (range). AN-R; anorexia nervosa-restricting type, AN-BP; anorexia nervosa-binge eating/purging type, BMI; body mass index, ECW; extracellular water, TBW; total body water

Three quarters of the patients were of the binge-eating/purging type (AN-BP), with a mean age of 44.05 ± 8.38 years—higher than the typical mean age of AN. The average BW, BMI, and percentage of body fat were 32.22 ± 3.8 kg, 13.12 ± 1.40 kg/m^2^, and 7.04 ± 4.55%, respectively. The weight restoration period was 302 days, which is longer than the length of an average hospital stay in Japan—26.3 days overall and 263.2 days for psychiatric beds, as reported by the Ministry of Health, Labour and Welfare [25]. Daily caloric intake during the period of weight restoration was almost the same as the energy requirements of a healthy adult woman (1,858 ± 278 kcal/day) [26].

During treatment, 38 patients gained more than 5 kg of BW, and 25 patients achieved a BMI > 18.5 kg/m^2^ prior to leaving prison. However, one patient only gained 0.2 kg during her 651-day admission period.

### Excess energy required to gain weight

EE1 values were considered outliers for 2 of the 41 patients because they were over three times the 75th percentile value. Excluding these outliers, the average and median EE1 values were 12,776.8 (SD: 3,334.8) and 12,120 kcal/kg respectively, with large individual differences (range: 6,636–22,064 kcal/kg) and a coefficient of variation (CV) of 26.1%.

### Factors for weight gain

**Fig. 1:**
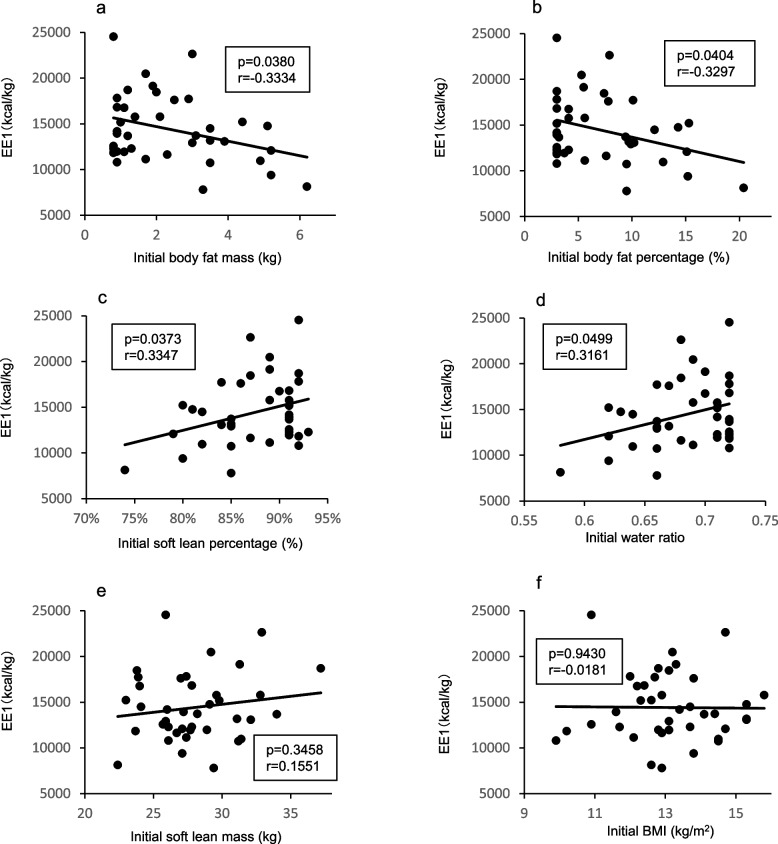
Relation of energy required for 1 kg weight gain to body composition. Plot of the relation between mean EE1 and (**a**) initial body fat mass, (**b**) body fat percentage, (**c**) soft lean percentage, (**d**) body water ratio, (**e**) soft lean mass, (**f**) initial BMI. EE1; excess energy to increase 1 kg body weight, BMI; body mass index

**Fig. 2:**
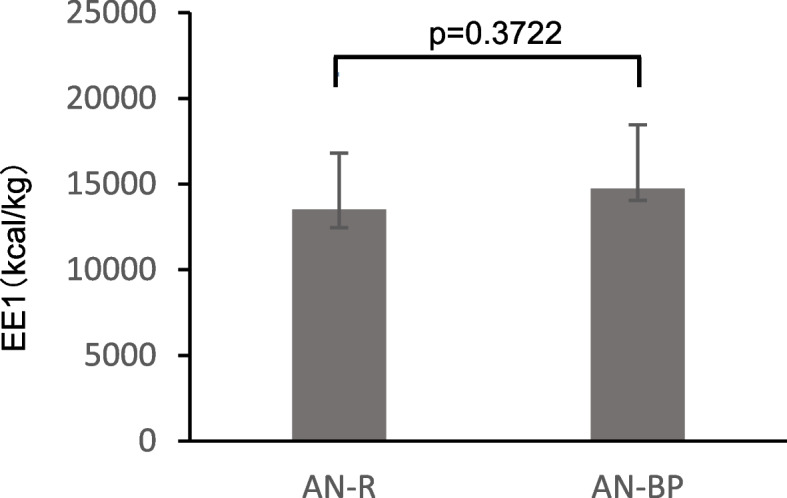
Relation of energy required for 1 kg weight gain to AN subtype

The relation between EE1 and the AN subtype and body composition were analyzed to elucidate factors that may cause weight gain. The relation between EE1, the AN subtype, and the first measured value of BMI, BFM, body fat percentage (BFP), soft lean mass, soft lean percentage (SLP), and body water ratio (BWR: body water/body weight) are shown in Figs. 1 and 2. A significant correlation was observed between EE1 and BFM (Fig. 1a), BFP (Fig. 1b), SLP (Fig. 1c), and BWR (Fig. 1d), while SLM (Fig. 1e) appeared to have no correlation with EE1. Furthermore, no significant differences in EE1 among the AN subtypes (Fig. 2) or initial BMI (Fig. 1g) were noted. In addition, a positive correlation was observed between the amount of weight gain and the length of the admission period (data not shown).

Secondly, we examined how the factors correlating with EE1 affected weight gain. Each item that correlated with EE1 was divided into two groups based on initial values, and the results of the comparison of the mean EE1 of each group are shown in Table 2**.** When divided into two groups, all items correlating with EE1 had a point at which a significant difference in EE1 was noted, with the mean EE1 in the groups with BFM ≤ 3 kg (*p* = 0.003) (Table 2), BFP ≤ 8% (*p* = 0.008) (Table 3), SLP > 85% (*p* = 0.011) (Table 4), and BWR > 0.665 (*p* = 0.011) (Table 5) higher than that of the other groups.

**Table 2:** Comparison of the mean excess energy to increase 1 kg body weight for each group. body fat mass

initial BFM (kg)	N	EE1 (kcal/kg)	*p*
≦2.5	24	13,565	0.0610
> 2.5	15	11,516	
≦3.0	27	13,800	0.0028
> 3.0	12	10,473	
≦3.5	32	13,264	0.0496
> 3.5	7	10,549	
≦4.0	33	13,200	0.0619
> 4.0	6	10,447	

EE1; excess energy to increase 1 kg body weight, BFM; body fat mass

**Table 3:** Comparison of the mean excess energy to increase 1 kg body weight for each group. body fat percentage

initial BFP (%)	N	EE1 (kcal/kg)	*p*
≦7.0	21	13,475	0.1607
> 7.0	18	11,962	
≦8.0	25	13,816	0.0075
> 8.0	14	10,921	
≦9.0	25	13,816	0.0075
> 9.0	14	10,921	
≦10.0	30	13,200	0.1504
> 10.0	9	11,366	

BFP; body fat percentage

**Table 4:** Comparison of the mean excess energy to increase 1 kg body weight for each group. soft lean percentage

initial SLP (%)	N	EE1 (kcal/kg)	*p*
≦84	7	10,798	0.0829
> 84	32	13,210	
≦85	13	10,896	0.0107
> 85	26	13,718	
≦86	15	11,249	0.0216
> 86	24	13,732	
≦87	17	11,499	0.0335
> 87	22	13,765	
≦88	18	11,962	0.1607
> 88	21	13,475	

SLP; soft lean percentage

**Table 5:** Comparison of the mean excess energy to increase 1 kg body weight for each group. body water ratio

initial BWR	N	EE1 (kcal/kg)	*p*
≦0.655	7	10,798	0.0829
> 0.655	32	13,210	
≦0.660	8	10,599	0.0365
> 0.660	31	13,339	
≦0.665	13	10,896	0.0107
> 0.665	26	13,716	
≦0.670	15	11,249	0.0216
> 0.670	24	13,732	
≦0.675	15	11,249	0.0216
> 0.675	24	13,732	
≦0.680	18	11,962	0.1607
> 0.680	21	13,475	

BWR; body water ratio

No correlation between EE1 and BW, age, total body water (TBW), intracellular water, extracellular water (ECW), or ECW/TBW was observed (data not shown).

## Discussion

This study, conducted in a long-term in-patient controlled setting where patients could not purge or engage in hyperactivity, examined the energy requirements and factors influencing the weight recovery of patients with AN. Our results suggest that patients with AN require greater energy intake to gain weight than do healthy individuals [27]. Furthermore the amount of energy intake required for patients with AN to gain weight varied considerably, and a body composition factor, the ratio of BFM to SLM prior to renourishment, affected the energy requirement.

Weight recovery is one of the core principles and the first goal of AN treatment. However, there are conflicting reports on the amount of energy required to gain 1-kg BW for patients with AN [8], which may explain inconsistencies in the current guidelines regarding the amount of calories required for weight recovery in these patients.

The current study showed that these patients with AN needed 12,777 kcal to gain 1 kg during the weight recovery period. According to a previously reported study on overfeeding [27], approximately 10,500 kcal of energy is needed to gain 1-kg BW in healthy individuals, indicating that patients with AN require more energy than healthy individuals to gain BW. Among studies that directly compared the amount of energy required to gain weight in patients with AN with that required for healthy controls, Forbes et al. reported that healthy controls needed more energy than patients with AN (AN, 4,730 kcal/kg; control, 7,090 kcal/kg); however, the AN group consisted of seven women and one man, while the control group consisted of only men (n = 17). Furthermore, only three individuals from the control group and the AN group were directly compared [28]. These factors may have caused the inconsistencies with our results.

Although it cannot be directly compared with the excess energy required for weight gain, resting energy expenditure (REE) was reported to increase during weight gain in both healthy participants and patients with AN. Furthermore, the increase in REE in healthy patients was proportional to the increase in FFM, while the increase in REE in patients with AN was disproportionately greater than the increase in FFM [29, 30]. There are large individual differences in the energy required for weight gain in healthy persons, which may be related to factors such as hereditary characteristics [31] and non-exercise activity thermogenesis (NEAT) [32, 33]. The present study was conducted in a prison setting where behavior and activity were strictly monitored and unnecessary physical activity beyond the set restrictions was controlled. Hyperactivity is a known symptom of AN, and it cannot be ruled out that some of our participants may have evaded supervision while engaged in physical activity. An increase in NEAT may have affected the increase in EE1. Furthermore, diet-induced thermogenesis (DIT) is also a factor that can affect energy expenditure. DIT is said to account for approximately 10% of energy consumption and varies between individuals [34], which may account for individual differences in EE. It has also been reported in AN that DIT is greater with increased energy intake, which may explain how EE is greater in patients with AN than in healthy women [16].

Our group has previously reported gut dysbiosis in patients with AN [12]. Also, germ-free mice transplanted with feces from patients with AN showed a decrease in BW gain and nutritional efficiency compared with mice transplanted with feces from healthy donors [35]. It has been speculated in the livestock industry that intestinal bacteria influence weight gain and loss, with antibiotics in livestock shown to produce weight gain and antibiotics in germ-free animals not producing this weight gain effect [36]. These reports indicate that abnormalities in the gut microbiota of patients with AN may be related to the energy cost of weight gain.

Several studies have investigated the amount of energy required to gain weight, with results varying widely among the studies and among participants in the same study [9–11, 28, 37–40]. Our results showed that the energy cost to gain 1-kg BW was 12,777 kcal/kg, higher than that reported in most previous studies, but relatively similarly to the values observed by Yamashita et al. [39]. Some differences exist between our study and that of the Yamashita group, which included patients in a general hospital setting and measured body composition using dual-energy X-ray absorptiometry. Nonetheless, the following similarities should be noted: 1) all participants were Japanese; 2) the observation period was longer than that in other studies (current study, mean 302 days; Yamashita et al. study, mean > 112 days; Gentile et al. study, mean 90 days); 3) the intake of participants was based on the daily requirements of a healthy woman, while caloric intake was greater than that required by healthy women in other studies (Russell et al., 3500–5500 kcal/day and Gentile et al., 1199–2935 kcal/day). The aforementioned factors may explain inconsistencies between the results of the present study and previously reported studies.

As previously noted, the amount of energy required to gain 1 kg during the weight recovery period of patients with AN has varied greatly among participants in the same study. In previous studies with more than 10 patients, the CV ranged from 25.4% to 95.4%, with a smaller number indicating a smaller degree of variability [10, 11, 37, 39]. The CV in our study was 26.1%, indicating that the amount of energy required to gain 1 kg varied among participants, excluding outliers, although by a smaller amount than that reported in other studies.

Regarding influencing factors, one study reported that patients with AN restricting-type (AN-R) required less energy for weight gain than patients with AN binge-eating/purging type (AN-BP) [10], while another study reported opposite results [41]. Additionally, patients with BFM < 4 kg or BMI < 14 kg/m^2^ prior to refeeding required less energy for weight gain compared with those with BFM ≥ 4 kg or BMI ≥ 14 kg/m^2^ [39]. In our study, we found a relation between EE1 and BFM, FMP, SLP, and BWR, although the subtype of AN and BMI did not appear to be associated with EE1. EE1 was significantly higher in patients with initial FM ≤ 3.0 kg, FP ≤ 8.0%, SLP > 85%, and BWR > 0.665 values, suggesting that the amount of energy required to gain weight may depend on the ratio of BFM to SLM (i.e., how undernourished or hyperactive the patient is), rather than on the patient’s physique. The ratio of BFM to SLM can serve as an indicator of nutritional status. A low BFM-to-SLM ratio, which reflects a low body fat percentage and high lean mass percentage, typically indicates a malnourished state in which fat stores are severely depleted while the proportion of metabolically active organs and muscle remains relatively high [3, 18]. In such cases, the resting energy expenditure per unit of body weight tends to increase because of the greater contribution of energy-demanding lean tissues such as the liver, kidneys, heart, and brain. Consequently, patients with a low BFM-to-SLM ratio may require significantly more energy for weight restoration than do individuals with a more balanced body composition [17]. During weight recovery, patients with this body composition may be particularly vulnerable to protein catabolism if their energy intake is not adequate. Therefore, although the proportion of carbohydrates should be reduced in the very early phase of nutritional rehabilitation [42], it may be beneficial to prioritize energy intake from fats and carbohydrates to suppress protein breakdown and support vital organ function. Once energy balance is restored, increased intake of high-quality protein and essential amino acids may help promote the recovery of FFM.

Considering that the participants in the present study included older and undernourished individuals, the effects of sarcopenia should also be considered, particularly in terms of re-nutrition and rehabilitation. Only three patients in our study population (all in their 40s) had an SMI < 5.7 kg/m^2^ that met the diagnostic criteria (Asian Working Group for Sarcopenia 2019) for sarcopenia. However, by approximately the end of the initial month, these patients no longer met the diagnostic criteria; therefore, the overall impact of sarcopenia was not considered.

The inconsistencies between our results and those of previous studies may be explained as follows: 1) the number of participants in our study was larger than that in previous studies, 2) our participants were older than those in previous studies, and 3) the setting for our study was a medical prison where strict observation conditions could be maintained. In a previous study that did not investigate the energy required to gain weight, anxiety, abdominal pain, activity level, and smoking were reported as factors that increase REE in patients with AN [8]. An increase in the actual REE may have increased the resultant energy value for weight gain, as the REE in our study was determined based on Scalfi’s formulation. We did not investigate anxiety or abdominal pain, and our participants did not smoke because it is prohibited in medical prisons in Japan. However, activity levels and NEAT may have affected our results. Patients with hyperactivity may experience increased NEAT and require more energy, even if their activity levels are restricted. To better evaluate the amount of energy required for weight gain, activity meters or metabolic rooms are essential.

The strengths of the current study include the medical prison setting, which allowed us to observe the participants for a longer period of time and under more strictly controlled conditions than those in place in other hospitals in Japan. Considering the typical traits of patients with AN, this may be an important factor in accurately investigating the number of calories consumed. Furthermore, this study included a larger number of participants than did previous studies.

The current study also has several limitations. First, we cannot exclude the possibility of a gap between the calculated total energy expenditure (TEE) and actual TEE values because we did not use an indirect calorimeter or an activity meter. Second, the participants were older than the average patient with AN; hence, our results may not be applicable to patients in their teens or twenties, which is the average age for patients with AN. Third, because Scalfi's formula is designed for women under the age of 30 years, it may not be suitable for use in patients aged 30 and above. Nonetheless, we decided to use this formula because there is no alternative well-established formula for the calculation of BMR in patients with AN aged 30 and above. Fourth, although receiver operating characteristic curve analysis is a commonly used method to determine optimal cutoff values, it was not employed in the present study due to the limited sample size. Previous studies suggest that at least 100 cases, with sufficient representation in each comparison group, are needed to ensure the statistical reliability and validity of receiver operating characteristic-derived thresholds [43, 44]. Given the relatively small number of participants in our study, we deemed that subgroup comparisons within the interquartile range would be a more appropriate and reliable approach for exploring variable associations. Fifth, because metabolism, gut microbiota, and other factors are involved in weight gain in AN, food intake during the plateau period may also play an important role. Therefore, to elucidate the specific factors contributing to weight gain, it would be desirable to perform an analysis that incorporates caloric intake not only during the weight gain phase but also during the plateau and weight loss phases. However, because this study included participants who did not reach a plateau period during their hospitalization, we focused solely on the weight gain phase and excluded data from the weight stabilization and weight loss phases following the attainment of a healthy weight. Further research, including studies with larger sample sizes, is needed.

## Conclusions

Patients with AN required approximately 12,800 kcal of “excess energy” for a 1-kg BW gain, although individual differences were considerable. Furthermore, severe malnutrition and hyperactivity in patients may lead to a greater energy requirement in order to gain weight. Providing greater energy intake to patients with severe cases of AN, with individual adjustments based on weight gain progress in the renourishment period, is critical.

## Data Availability

The datasets generated and/or analyzed during the current study are not publicly available due to privacy or ethical restrictions but are available from the corresponding author on reasonable request.

## Abbreviations

AN: Anorexia nervosa
BIA: Multifrequency bioelectrical impedance analysis
BW: Body weight
EE: Excess energy
EE1: Excess energy to increase 1 kg BW
BMI: Body mass index
AN-BP: Binge eating / purging type of anorexia nervosa
CV: Coefficient of variation
BFM: Body fat mass
BFP: Body fat percentage
SLM: Soft lean mass
SLP: Soft lean percentage
BWR: Body water ratio
TBW: Total body water
ECW: Extracellular water
REE: Resting energy expenditure
FFM: Free fat mass
NEAT: Non-Exercise Activity Thermogenesis
AN-R: Restricting-type of anorexia nervosa
